# Modeling and Error Compensation for Concentric Grinding of Spherical Surfaces

**DOI:** 10.3390/mi17070812

**Published:** 2026-07-05

**Authors:** Baozhen Li, Keyan Song, Dongxu Wu, Lin Sun, Yunfei Li

**Affiliations:** 1 Genertec Machine Tool Engineering Research Institute Co., Ltd., Beijing 100102, China; libaozhen1@gt.cn (B.L.); wudongxu@jcs.gt.cn (D.W.); liyunfei2@jcs.gt.cn (Y.L.); 2 Genertec Joint Research Institute, Xi’an Jiaotong University, Xi’an 710049, China; 3 School of Mechanical Engineering, Xi’an Jiaotong University, Xi’an 710049, China; handles@stu.xjtu.edu.cn

**Keywords:** optical components, spherical surface, grinding wheel, ultra-precision grinding

## Abstract

To improve the form accuracy of spherical surfaces generated by cup-wheel grinding, this paper presents a geometric modeling and error compensation method for concentric grinding of spherical surfaces. A cup-shaped arc grinding wheel, hereafter referred to as a cup wheel, is used as the grinding tool. The relative motion between the cup wheel and the workpiece is formulated so that the contact arc center of the wheel follows a trajectory that is concentric with the target spherical surface. Based on this principle, trajectory models for both convex and concave spherical surfaces are established, and the geometric constraints for cup-wheel dimension selection are analyzed. To compensate for tool-setting errors and wheel-wear-induced deviations, a central-peak-based error compensation model is further developed. Grinding experiments on a convex spherical sample were conducted to verify the proposed trajectory and compensation models. The results show that the form error PV value was reduced from 57.7 μm to 0.3 μm after compensation, demonstrating the effectiveness of the proposed model in improving spherical form accuracy.

## 1. Introduction

As a precision optical component and critical mechanical part, the spherical surface has irreplaceable application value in fields such as aerospace, medical devices, semiconductor manufacturing, and advanced optical systems [1,2]. For example, in high-performance imaging systems, high-precision spherical lenses are not only core components for achieving outstanding optical performance but also the foundation for fabricating complex optical surfaces such as aspheric mirrors. As modern industries continue to raise their demands on product performance, integration, and reliability, greater challenges are being posed to the precision, efficiency, and surface quality of spherical lens manufacturing.

The high hardness and brittleness of optical components pose significant challenges to machining, making efficient and precise processing a key area of research [3,4]. Traditional machining methods, such as turning and milling, have limitations in terms of precision and tend to cause surface damage when machining hard and brittle materials [5]. Although polishing can achieve extremely high surface finish, it suffers from low processing efficiency and makes implementing rapid compensation for surface form difficult [6]. As a high-precision and high-efficiency machining technique, grinding has attracted widespread attention in the field of precision spherical surface machining due to its ability to remove material in minute quantities, deliver outstanding surface integrity, and ensure superior form accuracy [7].

Multi-axis CNC machine tools comprise a key technology for the precision machining of complex surfaces [8]. Multi-axis linkage enables complex relative motions between the grinding wheel and the workpiece, allowing for precise control of the material removal process to generate high-precision spherical surfaces [9]. However, in practical grinding applications, the cumulative effects of factors such as machine geometric errors [10], tool wear [11], thermal deformation [12], and setup errors [13] often compromise machining accuracy and induce surface defects. Consequently, research on efficient trajectory planning and robust error compensation strategies is critical for enhancing the quality of spherical grinding.

Regarding the five-axis grinding of curved surfaces, researchers have investigated various surface generation methods utilizing different types of grinding wheels. Specifically, for the envelope grinding of spherical surfaces using cup wheels, the unique wheel geometry and generation principle enhance grinding efficiency. However, this approach imposes stringent requirements on tool path planning, necessitating precise control over the relative motion between the grinding wheel and the workpiece to ensure the uniformity and consistency of material removal. Xu [14] investigated a swing grinding process based on the equivalent sphere concept and a high-stiffness machine tool architecture with reduced axes. This work achieved high-efficiency multi-point contact grinding for large axisymmetric aspheric and spherical surfaces, thereby increasing the material removal rate while reducing machine complexity. Jiang [15] conducted research on tool path planning for small-aperture aspheric surfaces based on the arc envelope principle, effectively suppressing the mid-spatial frequency errors associated with traditional point-to-point grinding and enhancing surface generation quality. Yang [16] focused on the design of an elliptical toroidal grinding wheel and an adaptive path generation method for optical freeform surfaces, realizing high-efficiency and high-precision direct grinding of complex non-rotationally symmetric freeform surfaces.

In terms of error compensation, the profile accuracy of the grinding wheel dictates the machining precision of the workpiece. Excessive profile error in the dressed wheel directly results in non-conforming workpiece accuracy [17]. To maintain the wheel profile, researchers have explored various dressing methods for arc-shaped grinding wheels, including mechanical dressing [18,19], laser dressing [20,21], and electrical discharge machining (EDM) dressing [22,23]. Gu [24] proposed an error compensation method for single-point inclined-axis grinding that accounts for grinding wheel wear. By incorporating wheel wear factors and utilizing a modified polynomial equation to generate a new compensation trajectory, the profile error of optical aspheric surfaces was reduced to less than 150 nm.

In this work, the focus is placed on the modeling and error compensation of concentric grinding for spherical surfaces using a cup wheel on a cradle-type five-axis machine tool. First, geometric trajectory models are established for convex and concave spherical surfaces, describing the kinematic relationship between the cup wheel, the workpiece, and the cradle motion. Second, the feasible geometric conditions for cup-wheel dimension selection are analyzed to avoid wheel–workpiece interference. Third, an error compensation method based on the central peak feature is proposed to correct tool-setting errors and wheel-wear-induced trajectory deviations. Finally, a convex spherical grinding experiment is conducted to verify the effectiveness of the proposed trajectory and compensation models in improving form accuracy.

## 2. Method

### 2.1. Concentric Grinding Method for Spherical Surfaces

**Figure 1:**
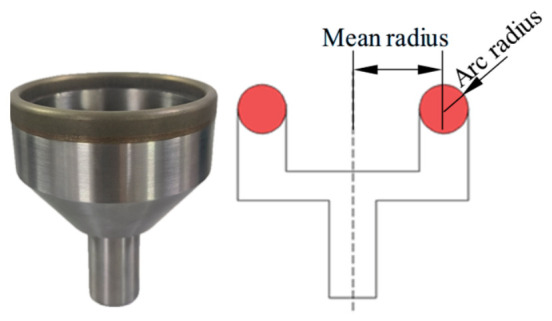
Cup-shaped grinding wheel.

Spherical grinding typically employs two main principles: form grinding and generation (envelope) grinding. Form grinding is relatively direct, involving dressing the grinding wheel into an arc shape that matches the spherical curvature to grind the workpiece directly. This method requires extremely high precision in wheel dressing. Generation grinding is a more common and flexible method, the core principle of which is using motion trajectories to envelop and form the spherical surface. The cup wheel and the workpiece are installed at a specific angle (axes intersecting). When the wheel rotates at high speed for grinding while the workpiece rotates around its own axis, the grinding point on the wheel forms a series of circular trajectories on the workpiece surface. By controlling the feed motion of the wheel along the axial direction, these trajectories cover the entire spherical surface, ultimately enveloping a sphere. This study utilizes a cup wheel as the grinding tool, as shown in Figure 1.

**Figure 2:**
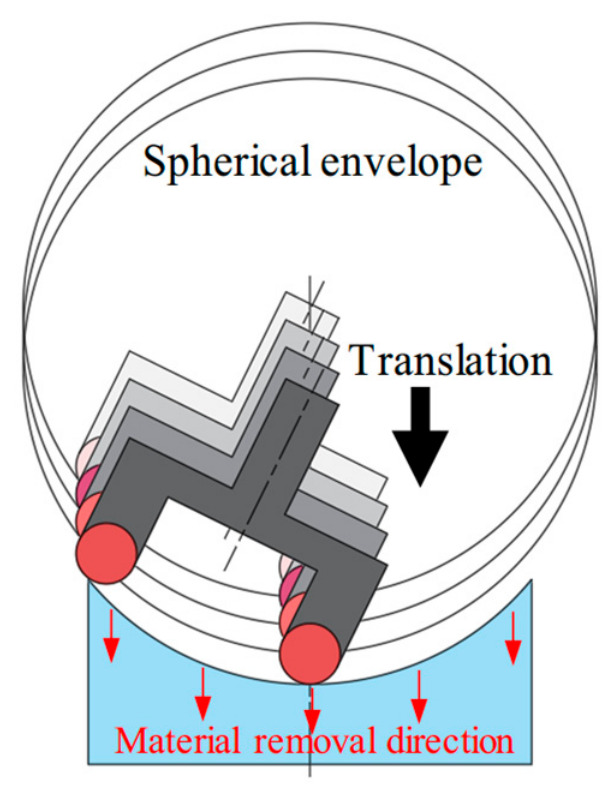
Traditional spherical grinding method.

Traditional spherical grinding methods involve controlling the axis of the cup wheel to form an angle with the cylindrical workpiece axis, followed by feeding the wheel toward the workpiece via two-axis linkage, as shown in Figure 2. In this method, the tool feed direction and the material removal direction are along the axis of the cylindrical workpiece, perpendicular to the top surface. Therefore, the normal component of the programmed infeed varies with the aperture position on the spherical surface. Near the pole, the feed direction is close to the surface normal, whereas, near the edge region, a larger tangential component is introduced. This geometric inconsistency may cause non-uniform nominal contact conditions over the spherical aperture.

**Figure 3:**
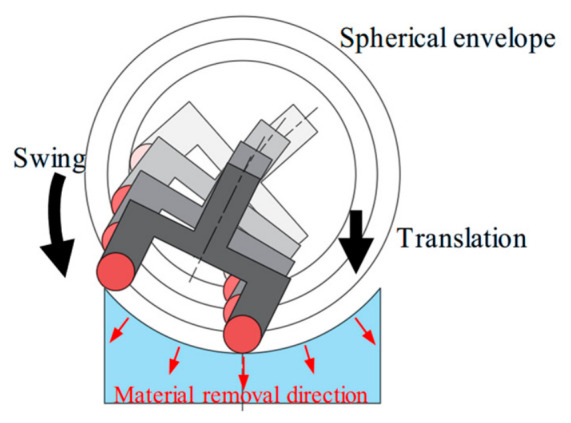
Concentric grinding method.

To establish a more geometrically consistent trajectory model for spherical grinding, a concentric grinding model is proposed in this study. As shown in Figure 3, the angle between the cup wheel axis and the cylindrical workpiece axis changes as the grinding process progresses. This method is achieved by controlling one rotary axis and two linear axes of the machine tool in a three-axis linkage. The tool feed direction is along the surface normal pointing toward the sphere center. From a geometric point of view, this motion mode makes the programmed feed direction consistent with the local normal direction of the spherical surface. Therefore, the nominal radial infeed can be kept more consistent over the spherical aperture than in conventional axial-feed grinding. It should be noted that the actual grinding force and material removal rate are also affected by contact pressure, sliding velocity, abrasive condition, coolant, workpiece material, and machine stiffness. Therefore, the present study focuses on trajectory modeling and form-error compensation, while direct force and surface-integrity evaluation will be investigated in future work.

### 2.2. Geometric Conditions for Cup Wheels

**Figure 4:**
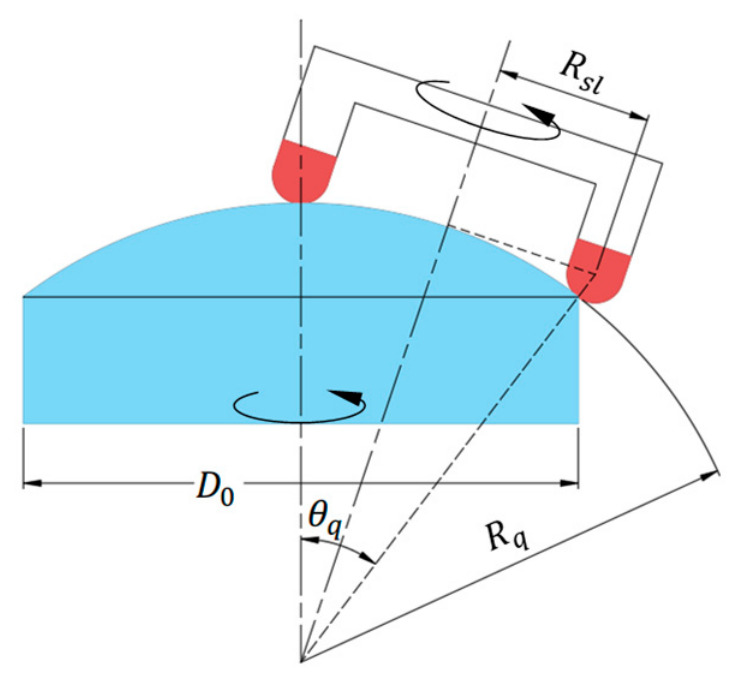
Minimum cup mouth size for grinding convex spherical surfaces.

Since spherical grinding is based on the envelope principle, the cup wheel and the spherical workpiece must be matched. Therefore, based on the dimensions of the spherical surface to be ground, there are specific requirements for the wheel dimensions. The method proposed in this paper allows for the use of a range of cup wheel sizes to machine a range of spherical sizes. As shown in Figure 4, for machining a convex spherical surface, the minimum allowable diameter for the cup wheel must satisfy the condition where the lip of the cup just touches the edge of the workpiece spherical surface. At this point,

(1) $$R_{sl\min}=(R_{q}+r_{sl})\sin\frac{\theta_{q}}{2}$$

where $R_{sl\min}$ represents the minimum cup radius of the cup wheel, below which machining cannot be accomplished, $R_{q}$ represents radius of desired sphere, $r_{sl}$ represents small arc radius of the wheel edge (lip radius as shown in Figure 1), and $\theta_{q}$ represents the arc angle occupied by the spherical portion of the workpiece, a characteristic of the workpiece itself, satisfying $\sin\theta_{q}=D_{0}/2R_{q}$.

The symbols used in the following trajectory and compensation equations are defined when they first appear.

After completing the convex surface machining using this method, the maximum allowable diameter for the cup wheel corresponds to the condition where its inner diameter is exactly equal to the spherical surface diameter (i.e., the wheel can fit a hemisphere). At this point,

(2) $$R_{sl\max}=R_{q}+r_{sl}$$

where $R_{sl\max}$ represents the maximum cup radius of the cup wheel that can be used during machining.

**Figure 5:**
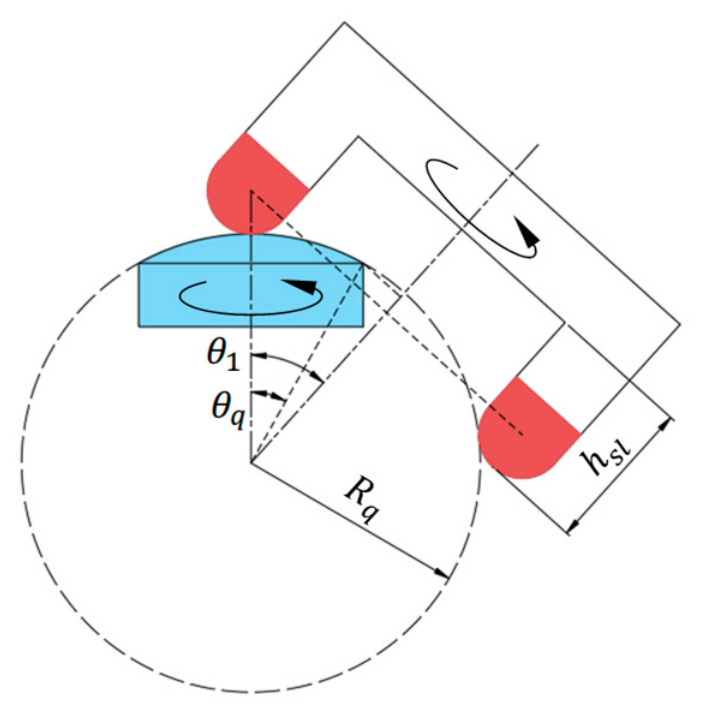
Small workpiece.

Furthermore, the cup section of the wheel serves to accommodate the workpiece. To avoid interference, the depth of the cup must meet certain requirements; specifically, it must not be lower than a certain value; otherwise, the bottom of the cup will collide with the workpiece during grinding. For a selected cup wheel with radius $R_{sl}$, the calculation of the cup depth depends on two scenarios:

For workpiece with $\theta_{q}<\theta_{1}$, referred to as small workpiece (Figure 5), the condition to avoid interference is that the bottom of the cup wheel does not touch the edge of the workpiece:

(3) $$h_{sl\min}>R_{q}\cos(\theta_{1}-\theta_{q})-(R_{q}+r_{sl})\cos\theta_{1}+r_{sl}$$

where $h_{sl\min}$ represents the minimum allowable depth of the cup wheel used, $\theta_{1}$ is the angle between the cup wheel axis and the workpiece axis at the end of grinding, satisfying $\sin\theta_{1}=R_{sl}/(R_{q}+r_{sl})$, and $R_{sl}$ represents cup radius of the cup-shaped arc wheel.

**Figure 6:**
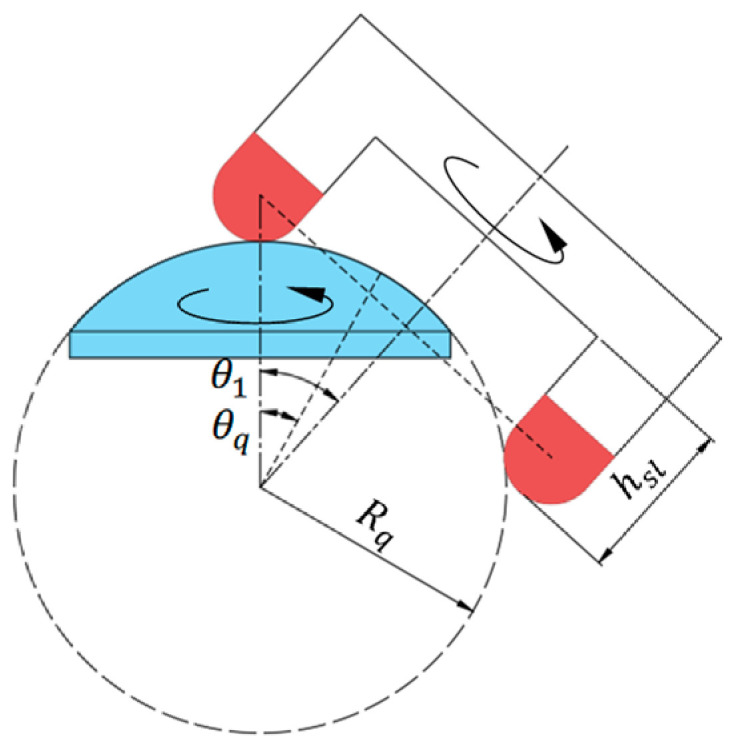
Large workpiece.

For workpiece with $\theta_{q}>\theta_{1}$, referred to as large workpiece (Figure 6), the condition to avoid interference is that the bottom of the cup wheel does not touch the spherical surface:

(4) $$h_{sl\min}>R_{q}-(R_{q}+r_{sl})\cos\theta_{1}+r_{sl}$$

**Figure 7:**
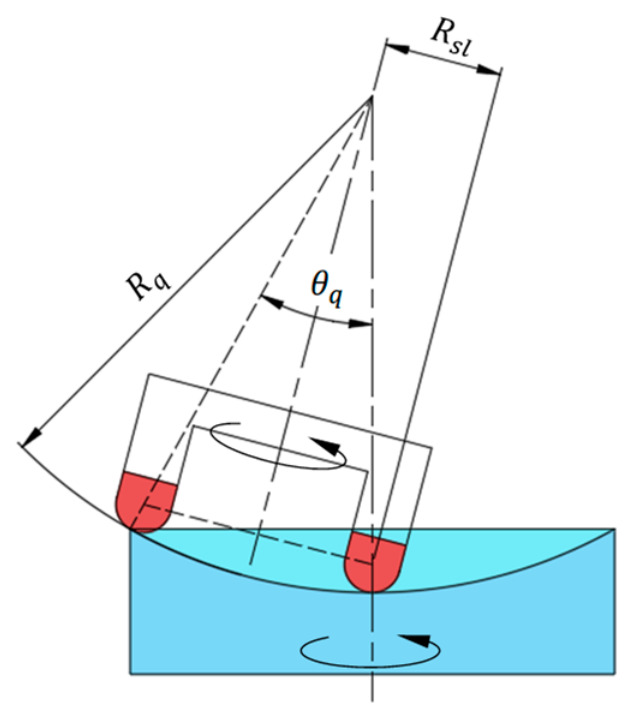
Minimum cup mouth size for grinding concave spherical surfaces.

Similarly, as shown in Figure 7, for machining a concave spherical surface, the minimum allowable diameter for the cup wheel corresponds to the condition where the cup lip just touches the edge of the spherical surface. Given $\sin\theta_{q}=D_{0}/2R_{q}$, then:

(5) $$R_{sl\min}=(R_{q}-r_{sl})\sin\frac{\theta_{q}}{2}$$

For concave surfaces, the maximum allowable diameter corresponds to the condition where the outer diameter of the wheel is exactly equal to the spherical surface diameter:

(6) $$R_{sl\max}=R_{q}-r_{sl}$$

The maximum allowable outer diameter of the cup wheel mentioned above corresponds to the theoretical case. In practice, due to the wide variety of possible workpiece fixture configurations and cup wheel geometries, it is recommended that the actual diameter of the cup wheel be selected slightly larger than the minimum value.

### 2.3. Cradle-Type Machine Grinding Method for Convex Surfaces

The equipment used in this study is a five-axis grinding machine tool with A–C axis linkage. When the machine tool is in A–C coupling mode, it can be regarded as equivalent to a cradle-type 5-axis machine tool. In this configuration, spherical grinding can be derived following the method of generating spherical surfaces using a cup wheel on a cradle-type 5-axis machine. In the method proposed in this study, the linkage motion is performed among the X/Y axes, Z axis, and A axis. In the A–C linkage mode of the equipment used, the C axis serves solely to maintain continuous high-speed rotation of the workpiece rather than controlling its orientation to a specific angular position.

**Figure 8:**
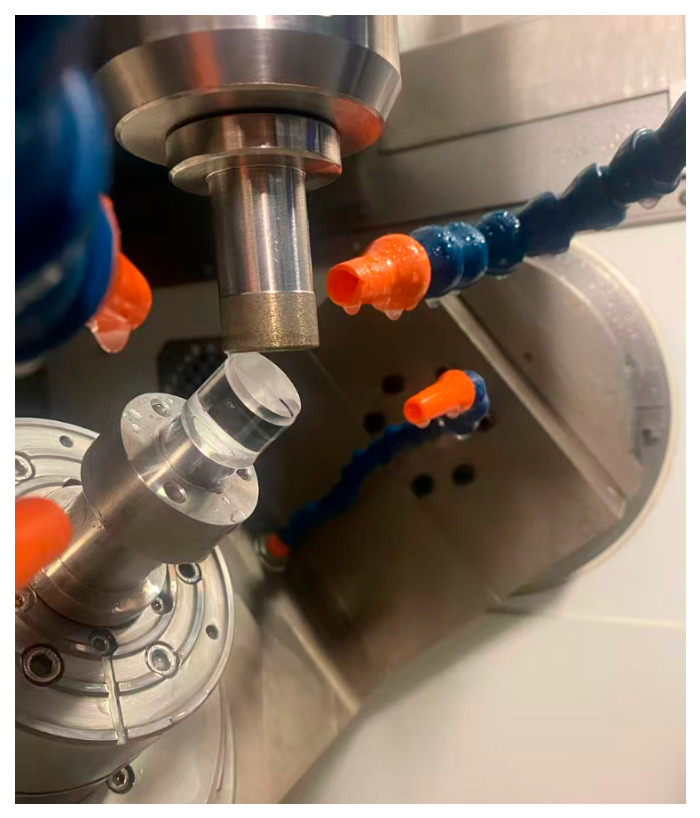
AC cradle-type five-axis machine tool.

As shown in Figure 8, when using a cradle-type 5-axis machine, the CNC system controls movement in X, Y, and Z directions, as well as rotation of A axis and C axis. By using the cup wheel and coordinating the two linear axes with one cradle axis (3-axis linkage), spherical grinding is achieved.

In a single grinding pass, the workpiece swings in the YOZ plane while rotating around its own axis. The cup wheel moves in the YOZ plane, synchronized with the spindle, while the spindle drives the wheel rotation. These combined rotational motions form an envelope spherical surface with a specific envelope radius. During the grinding process, the radius of the envelope sphere decreases uniformly over time. The material removal direction is always along the radius pointing toward the sphere center, and the removal thickness is uniform. After a set time t, the original cylindrical blank’s top surface becomes a spherical surface with the desired radius.

Figure 8 shows the setup where the cylindrical blank is mounted on the turntable and the cup wheel is on the spindle. Tool setting is performed based on wheel and workpiece dimensions. The axes are moved to initial positions, and rotation speeds for the turntable and spindle are set. By inputting the motion parameters for the X, Y, Z, and cradle axes and the grinding time t, the spherical surface is machined via the envelope trajectory. After time t, the cradle swings to angle $\theta_{1}$, and the cylindrical workpiece is ground into a spherical lens.

The derivation of the model assumes a cup wheel with a lip radius of $R_{sl}$ and a small edge arc radius of $r_{sl}$.

**Figure 9:**
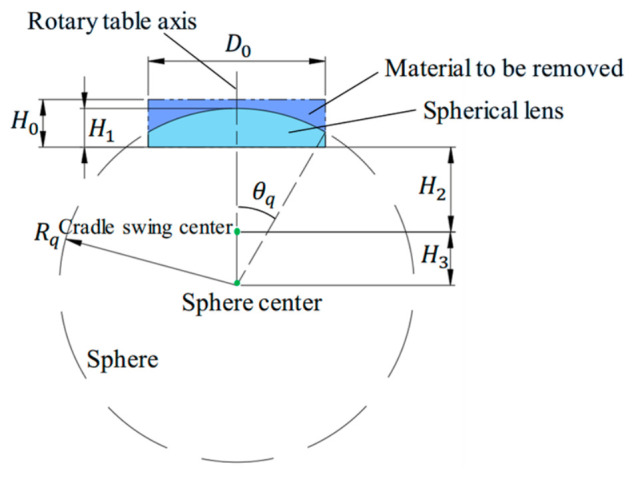
Convex spherical workpiece dimensions.

The convex workpiece is shown in Figure 9. The cylindrical blank has a diameter $D_{0}$ and height $H_{0}$. The desired convex lens has a center thickness $H_{1}$, spherical radius $R_{q}$, and an angle $\theta_{q}$ between the axis and the line connecting the sphere center to the edge. After mounting on the turntable, the distance between the workpiece bottom and the cradle swing center is $H_{2}$, and the distance between the cradle swing center and the sphere center is $H_{3}$.

(7) $$H_{3}=R_{q}-H_{1}-H_{2}$$

**Figure 10:**
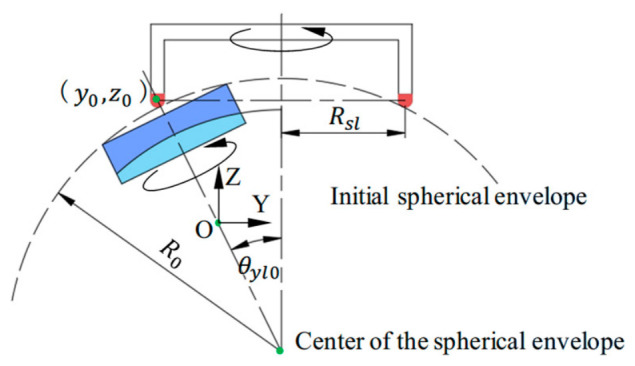
Initial position of the concave spherical surface.

The grinding process involves the linkage of the cradle axis with the Y and Z axes. The initial linkage position is shown in Figure 10. In the YOZ plane, point O is the cradle swing center. Taking the cradle swing center as the origin (0, 0), the coordinates of the cup wheel arc center at any moment are $\left(y_{yl},z_{yl}\right)$. The envelope sphere formed by the rotation of the wheel and workpiece has a radius R. At the initial position, the envelope radius is $R_{0}$. Since the formed sphere shares the same center as the target sphere, the following applies:

(8) $${R_{0}}^{2}={\left(R_{q}+H_{0}-H_{1}\right)}^{2}+{\left(\frac{D_{0}}{2}\right)}^{2}$$

where $R_{0}$ represents the initial envelope sphere radius, $R_{q}$ represents the required spherical radius, $H_{0}$ is the initial workpiece height, $H_{1}$ is the center thickness of the desired convex surface, and $D_{0}$ is the workpiece diameter.

The initial cradle swing angle is $\theta_{yl0}$:

(9) $$\sin\theta_{yl0}=\frac{R_{sl}}{R_{0}+r_{sl}}$$

Relative to the cradle swing center, the initial coordinates of the center of the contact arc of the cup wheel $\left(y_{yl0},z_{yl0}\right)$ are:

(10) $$\begin{array}{l} y_{yl0}=(R_{0}+r_{sl}-H_{3})\sin\theta_{yl0}\\ z_{yl0}=(R_{0}+r_{sl}-H_{3})\cos\theta_{yl0} \end{array}$$

**Figure 11:**
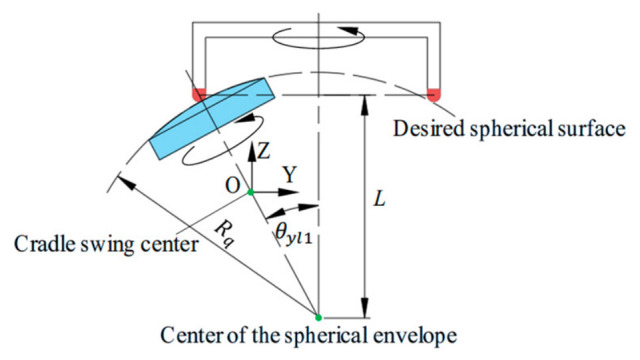
End position of the convex spherical surface.

As shown in Figure 11, after grinding is completed, the envelope sphere radius is $R_{q}$, and the cradle swing angle is $\theta_{yl1}$,

(11) $$\sin\theta_{yl1}=\frac{R_{sl}}{R_{q}+r_{sl}}$$

During the process, the envelope radius R decreases uniformly from $R_{0}$ to $R_{q}$ over time $t_{0}$. The rate of radius reduction $f_{convex}$ is:

(12) $$f_{convex}=\frac{R_{0}-R_{q}}{t_{0}}$$

At any time t, the envelope sphere radius R is:

(13) $$R=R_{0}-f_{convex}t$$

At same time t, the cradle swing angle *θ_yl_* satisfies:

(14) $$\sin\theta_{yl}=\frac{R_{sl}}{R+r_{sl}}$$

Combining Equations (9), (10), (13) and (14), the motion trajectory of the center of the cup wheel contact arc during convex grinding, with the cradle swing center as the origin, is derived as:

(15) $$\begin{array}{l} y_{yl}=(R_{0}-f_{convex}t+r_{sl}-H_{3})\sin\theta_{yl}\\ z_{yl}=(R_{0}-f_{convex}t+r_{sl}-H_{3})\cos\theta_{yl}\\ A_{yl}=\theta_{yl}=\arcsin\frac{R_{sl}}{R_{0}-f_{convex}t+r_{sl}} \end{array}$$

### 2.4. Cradle-Type Machine Grinding Method for Concave Surfaces

**Figure 12:**
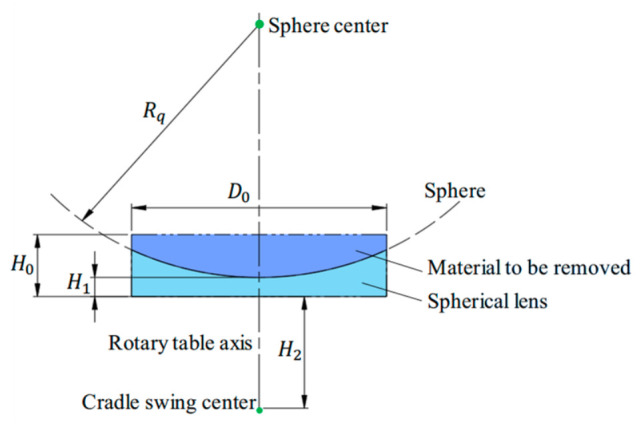
Dimensions of concave spherical workpieces.

For concave surfaces, combining the model used for convex surfaces, the workpiece is shown in Figure 12. The cylindrical blank has diameter $D_{0}$ and height $H_{0}$. The processed spherical workpiece has a center thickness $H_{1}$ and spherical radius $R_{q}$. After mounting, the distance between the workpiece bottom and the cradle swing center is $H_{2}$.

**Figure 13:**
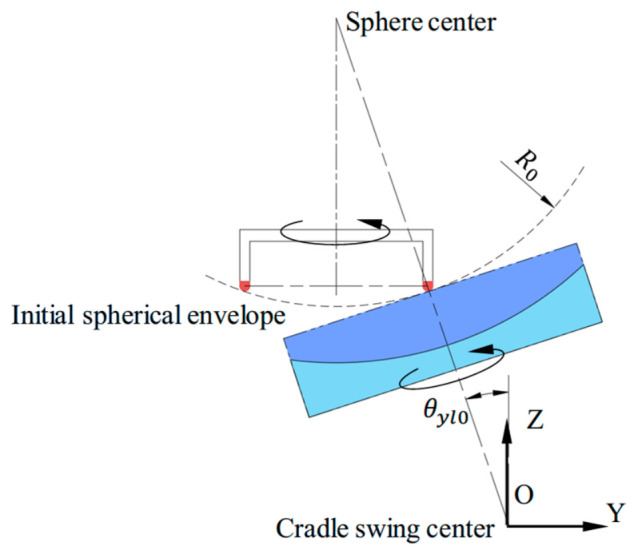
Initial position of the concave spherical surface.

The initial position is shown in Figure 13. The cradle swing angle is $\theta_{yl0}$. The cup wheel creates a circular grinding motion on the face, which, combined with the workpiece rotation, forms the envelope sphere. The initial envelope sphere radius $R_{0}$ satisfies:

(16) $$R_{0}=R_{q}-(H_{0}-H_{1})$$

(17) $$\sin\theta_{yl0}=\frac{R_{sl}}{R_{0}-r_{sl}}$$

Relative to the cradle swing center, the initial coordinates of the center of the cup wheel contact arc $\left(y_{yl0},z_{yl0}\right)$ are:

(18) $$\begin{array}{l} y_{yl0}=(H_{2}+H_{0}+r_{sl})\sin\theta_{yl0}\\ z_{yl0}=(H_{2}+H_{0}+r_{sl})\cos\theta_{yl0} \end{array}$$

**Figure 14:**
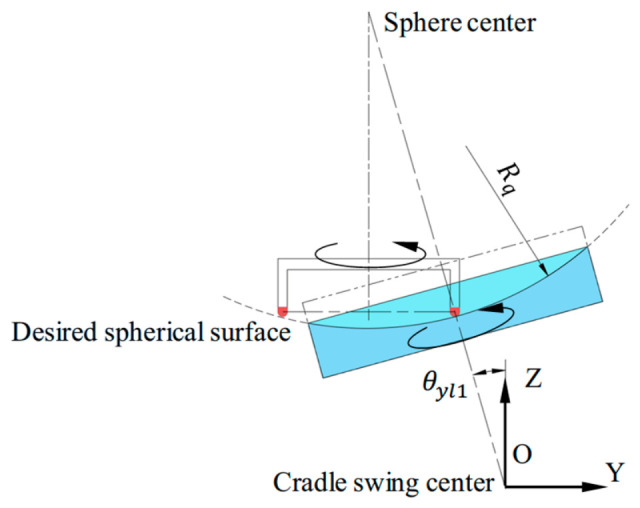
End position of the concave spherical surface.

The final position is shown in Figure 14. After grinding, the cradle axis is at angle $\theta_{yl1}$, and the envelope radius is exactly the target radius $R_{q}$. During the process, the envelope radius increases uniformly from $R_{0}$ to $R_{q}$ over time $t_{0}$. The rate of radius increase is $f_{concave}$:

(19) $$f_{concave}=\frac{R_{q}-R_{0}}{t_{0}}$$

At any time t, the envelope sphere radius R is:

(20) $$R=R_{0}+f_{convex}t$$

At same time t, the cradle swing angle θ satisfies:

(21) $$\sin\theta_{yl}=\frac{R_{sl}}{R-r_{sl}}$$

Combining Equations (16)–(18) and (20), the motion trajectory for concave grinding is derived as:

(22) $$\begin{array}{l} y_{yl}=(H_{2}+H_{0}-f_{concave}t+r_{sl})\sin\theta_{yl}\\ z_{yl}=(H_{2}+H_{0}-f_{concave}t+r_{sl})\cos\theta_{yl}\\ A_{yl}=\theta_{yl}=\arcsin\frac{R_{sl}}{R_{0}+f_{concave}t-r_{sl}} \end{array}$$

### 2.5. Error Compensation for Cradle-Type Grinding of Convex Surfaces

**Figure 15:**
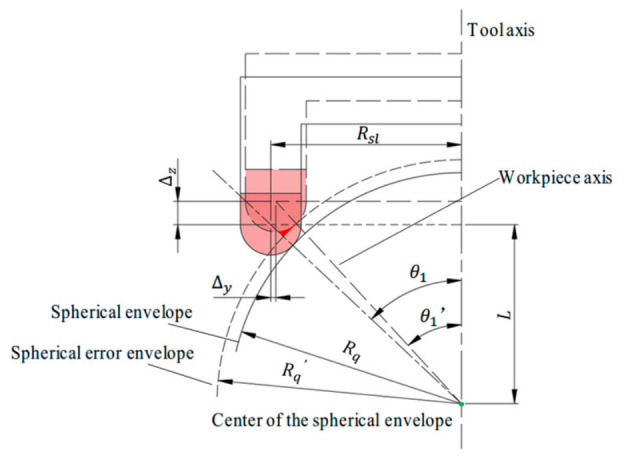
Sources of error in cradle-type grinding of convex spherical surfaces.

For convex surfaces, as shown in Figure 15, when there are tool setting or wear errors with the cup wheel, the center of the wheel arc deviates from the ideal position by Δy and Δz in the horizontal Y and vertical Z directions within the YOZ plane, respectively. Both deviations cause a change in the envelope sphere radius, ultimately affecting the actual ground spherical radius.

Considering Figure 11, in the ideal case, the Z-distance between the center of the cup wheel face diameter and the center of the envelope sphere after processing is *L*:

(23) $$L=\sqrt{{\left(R_{q}+r_{sl}\right)}^{2}-{R_{sl}}^{2}}$$

Based on Figure 15, when errors Δy and Δz exist, the Z-distance becomes $L^{\prime }=L+\Delta z$, and the envelope sphere radius becomes ${R_{q}}^{\prime }$:

(24) $${R_{q}}^{\prime }=\sqrt{{\left(R_{sl}+\Delta y\right)}^{2}+{\left(L+\Delta z\right)}^{2}}-r_{sl}$$

where ${R_{q}}^{\prime }$ represents the actual radius of the machined spherical surface, Δy represents the horizontal deviation of the cup wheel, and Δz represents its vertical deviation.

The angle between the wheel axis and the line connecting the wheel arc center to the sphere center becomes ${\theta_{1}}^{\prime }$:

(25) $$\begin{array}{l} \sin{\theta_{1}}^{\prime }=\frac{R_{sl}+\Delta y}{{R_{q}}^{\prime }+r_{sl}}\\ \cos{\theta_{1}}^{\prime }=\frac{L+\Delta z}{{R_{q}}^{\prime }+r_{sl}} \end{array}$$

**Figure 16:**
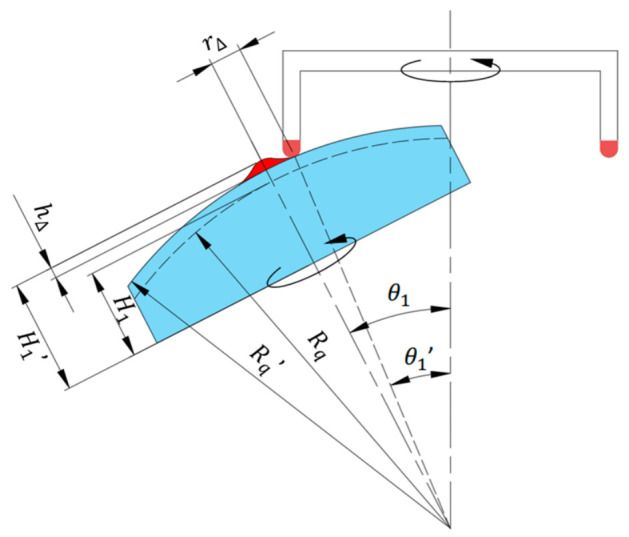
Characterization of convex spherical surface errors using cradle grinding.

As shown in Figure 16, when errors Δy and Δz are present, a central protuberance (peak) appears at the sphere center. The radius of this peak is $r_{\Delta }$, and the total height of the convex workpiece changes from $H_{1}$ to ${H_{1}}^{\prime }$.

Based on Figure 16, the radius of the central anomalous region is:

(26) $$r_{\Delta }={R_{q}}^{\prime }\sin[\pm (\theta_{1}-{\theta_{1}}^{\prime })]$$

Combining Equations (25) and (26), we obtain:

(27) $$\begin{array}{l} \Delta y=({R_{q}}^{\prime }+r_{sl})\sin(\arcsin\frac{R_{sl}}{R_{q}+r_{sl}}\pm \arcsin\frac{r_{\Delta }}{{R_{q}}^{\prime }})-R_{sl}\\ \Delta z=({R_{q}}^{\prime }+r_{sl})\cos(\arcsin\frac{R_{sl}}{R_{q}+r_{sl}}\pm \arcsin\frac{r_{\Delta }}{{R_{q}}^{\prime }})-L \end{array}$$

Compensation Procedure: After machining, measure the actual spherical radius ${R_{q}}^{\prime }$ and the radius of the central circular protuberance $r_{\Delta }$ on the workpiece. Calculate Δy and Δz using Equation (27). Incorporate Δy and Δz into the machining trajectory shown in Equation (15) to compensate. Perform the grinding process again until the surface form error of the workpiece is within the allowable range, thereby completing the spherical compensation.

### 2.6. Error Compensation for Cradle-Type Grinding of Concave Surfaces

**Figure 17:**
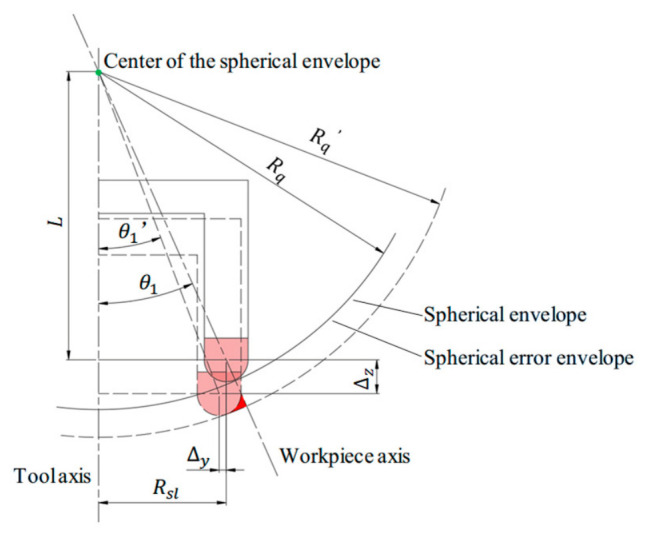
Sources of error in cradle-type grinding of concave spherical surfaces.

For concave surfaces, as shown in Figure 17, deviations Δy and Δz similarly cause changes in the envelope radius.

When errors Δy and Δz exist, the Z-distance becomes $L^{\prime }=L+\Delta z$, and the envelope sphere radius becomes ${R_{q}}^{\prime }$:

(28) $${R_{q}}^{\prime }=\sqrt{{\left(R_{sl}+\Delta y\right)}^{2}+{\left(L+\Delta z\right)}^{2}}+r_{sl}$$

The angle becomes ${\theta_{1}}^{\prime }$:

(29) $$\begin{array}{l} \sin{\theta_{1}}^{\prime }=\frac{R_{sl}+\Delta y}{{R_{q}}^{\prime }-r_{sl}}\\ \cos{\theta_{1}}^{\prime }=\frac{L+\Delta z}{{R_{q}}^{\prime }r_{sl}} \end{array}$$

**Figure 18:**
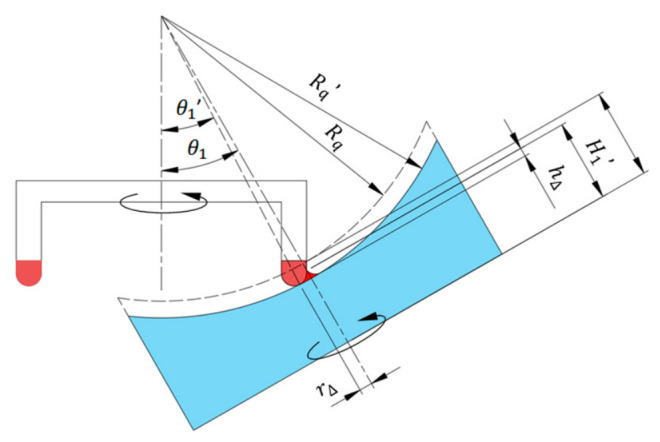
Characterization of concave spherical surface errors in cradle grinding.

As shown in Figure 18, a central peak appears with radius $r_{\Delta }$:

(30) $$r_{\Delta }={R_{q}}^{\prime }\sin[\pm (\theta_{1}-{\theta_{1}}^{\prime })]$$

Combining Equations (29) and (30):

(31) $$\begin{array}{l} \Delta y=({R_{q}}^{\prime }-r_{sl})\sin(\arcsin\frac{R_{sl}}{R_{q}-r_{sl}}\pm \arcsin\frac{r_{\Delta }}{{R_{q}}^{\prime }})-R_{sl}\\ \Delta z=({R_{q}}^{\prime }-r_{sl})\cos(\arcsin\frac{R_{sl}}{R_{q}-r_{sl}}\pm \arcsin\frac{r_{\Delta }}{{R_{q}}^{\prime }})-L \end{array}$$

Compensation Procedure: Similar to the convex case, measure the actual radius $R_{q}^{\prime }$ and the central peak radius $r_{\Delta }$. Calculate Δy and Δz using Equation (31). Apply these compensation values to the trajectory in Equation (22) and re-grind until tolerance is met.

The proposed trajectory and compensation models are geometry-based and parameterized by the designed spherical radius, workpiece diameter, cup radius, wheel lip arc radius, mounting distance, and cradle-center position. Therefore, the same modeling procedure can be applied to spherical workpieces with different radii and to cup wheels with different arc radii provided that the geometric interference constraints are satisfied. For each new wheel–workpiece combination, the initial envelope radius, final envelope radius, cradle swing angle, and Y–Z axis trajectory should be recalculated.

The compensation model is applicable when the dominant machining error can be represented by equivalent offsets of the cup-wheel contact arc center in the Y–Z plane. For very small spherical radii, the compensation sensitivity may increase because a small offset can cause a relatively large curvature deviation. For very large spherical radii, the central peak feature may become less obvious, which can increase measurement uncertainty. Furthermore, it can be observed from Equation (1) that, for workpieces with very large diameters, there may not be a correspondingly large cup wheel available to match them. These factors define the practical applicability range of the proposed compensation method.

## 3. Experiment

To verify the correctness of the proposed trajectory model and compensation model, this section utilizes a cup wheel to conduct grinding experiments on a convex spherical surface. The theoretical model is validated through a complete workflow of grinding, measurement, compensation, and re-measurement.

**Figure 19:**
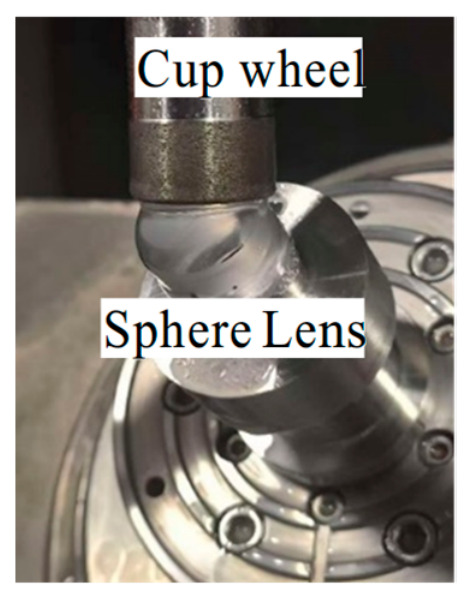
Spherical grinding process.

**Figure 20:**
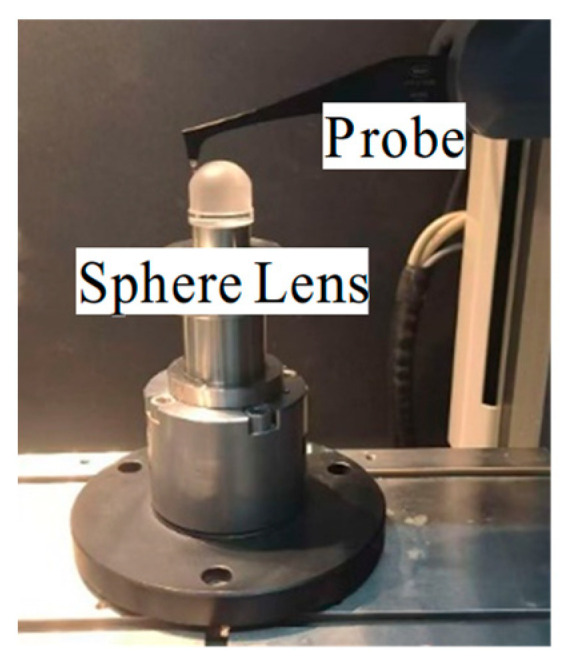
Spherical surface measurement.

Procedure:

1. Generate the grinding trajectory based on the theoretical model in Section 2.3.
2. Perform the convex grinding using a CNC machine tool (Figure 19).
3. Measure the ground workpiece using a Mahr profilometer (Figure 20) to obtain actual surface data.
4. Compare measurement data with the theoretical model, analyze the form error, and identify the central peak feature.
5. Based on the error analysis, calculate the compensation values using the proposed model, modify the grinding trajectory, and perform a second grinding pass and measurement to verify the compensation effect.

The tool and workpiece parameters are shown in Table 1.

**Figure 21:**
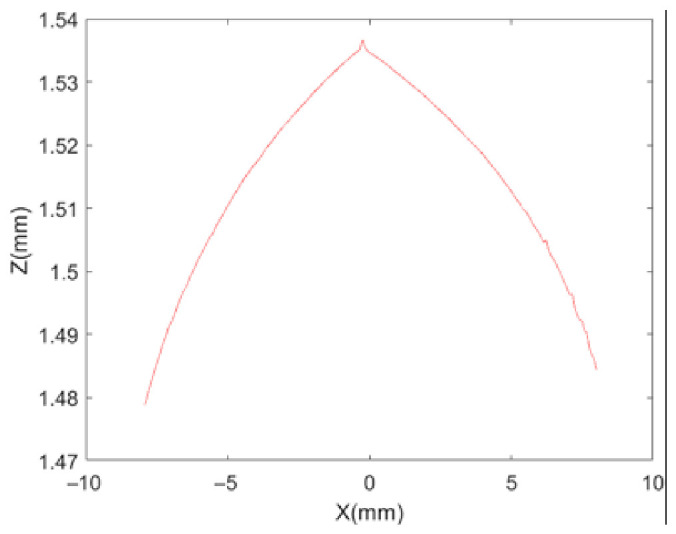
Spherical grinding surface form error.

**Figure 22:**
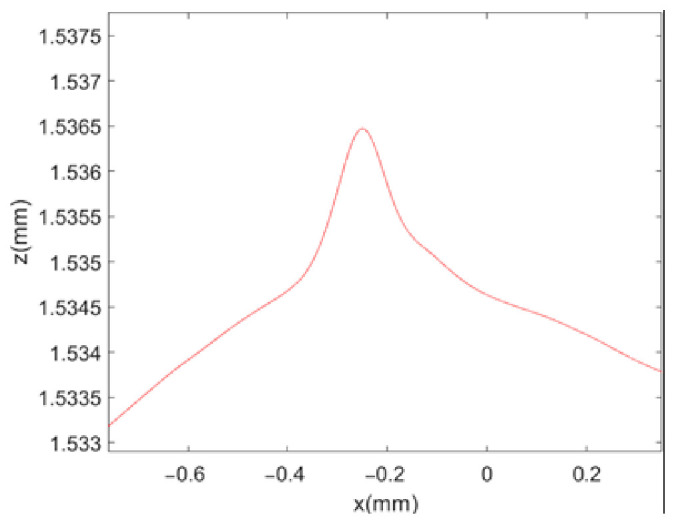
Spherical center peak.

Since the workpiece is rotationally symmetric, the measurement involves scanning a single line across the surface. The result is a set of x- and z-coordinates. Substituting the x-values from the measurement into the spherical equation yields the theoretical z-values. The difference between these and the measured z-values represents the form error at each x-coordinate, as shown in Figure 21. Magnifying the top section reveals a protrusion (Figure 22), which is the central peak caused by tool setting and wear errors. The PV value is 57.7 µm, and a distinct peak is visible at the top.

**Figure 23:**
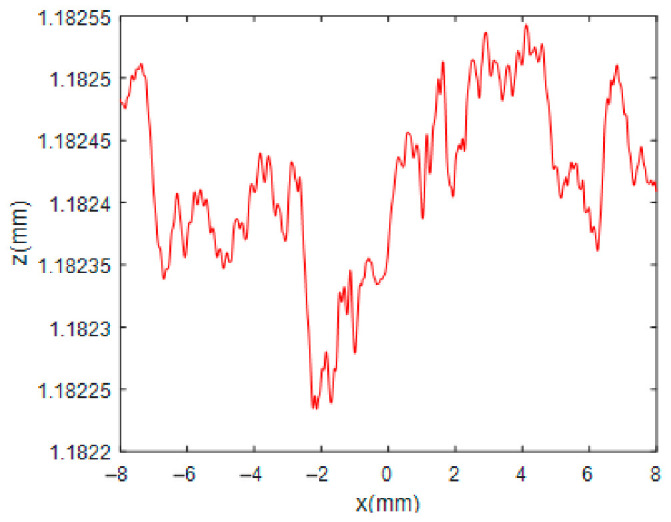
Spherical compensation effect.

By analyzing the x-coordinates, the radius of the area occupied by the peak, $r_{\Delta }$, is determined to be 0.1 mm. The Mahr profilometer measurement gives the actual spherical radius ${R_{q}}^{\prime }$ as 10.8756 mm. Using Equation (27), the compensation values are calculated as Δy=−0.167 mm and Δz=0.017 mm. These compensation values were added to the grinding trajectory for a compensation grinding pass. The form error after compensation is shown in Figure 23. The PV value was reduced to 0.3 µm, proving the effectiveness of the proposed compensation method. In addition, the surface roughness of the compensated spherical surface was measured, and the arithmetic mean roughness Ra was 0.08 μm. This result indicates that the proposed concentric grinding process can obtain a relatively smooth spherical surface under the tested machining conditions.

## 4. Conclusions

This study conducted systematic research on spherical machining trajectory planning and error compensation methods using cup wheels on five-axis linkage optical grinding machines. The main contributions are as follows:

A concentric grinding trajectory model was established for spherical surfaces. By describing the grinding process through the variations in a concentric envelope sphere, the proposed model provides a geometric basis for generating convex and concave spherical surfaces.

Wheel selection criteria were clarified. Based on the envelope grinding principle, the geometric relationship between the cup wheel and the workpiece was analyzed, providing selection criteria for the cup wheel diameter and cup depth for both convex and concave surfaces.

Complete grinding trajectories were derived for cradle-type machine configurations. Mathematical models for grinding convex and concave surfaces were established, enabling the cup-wheel contact arc center to follow the required concentric trajectory.

A spherical error compensation method was proposed. The typical central peak form error caused by wheel alignment or wear errors was deeply analyzed. A quantitative relationship model between the error magnitude and trajectory deviation was established for the AC machine configuration. Based on this, a compensation method was proposed that infers compensation values from the measured central peak error and corrects the original grinding trajectory.

A convex spherical grinding experiment was conducted to verify the proposed model. After one compensation cycle, the form error PV value decreased from 57.7 μm to 0.3 μm, demonstrating that the proposed compensation method can effectively improve spherical form accuracy under the tested conditions.

Future work will include comparative experiments with conventional envelope grinding, grinding force measurement, surface roughness evaluation, and subsurface damage analysis to further assess the influence of the proposed trajectory model on surface integrity.

## Figures and Tables

**Table 1:** Spherical grinding dimension table.

Cup Wheel		Workpiece	
Cup radius $R_{sl}$/mm	Arc radius $r_{sl}$/mm	Spherical radius $R_{q}$/mm	Workpiece diameter $D_{0}$/mm
10	1.37	11	22

## Data Availability

The datasets used or analyzed during the current study are available from the corresponding author upon reasonable request.
